# Effect of microabrasion on the staining susceptibility of enamel: An in vitro study

**DOI:** 10.34172/joddd.2022.016

**Published:** 2022-10-15

**Authors:** Hasibe Sevilay Bahadir, Merve Haberal, Çiğdem Çelik

**Affiliations:** ^1^Department of Restorative Dentistry, Faculty of Dentistry, Ankara Yıldırım Beyazıt University, Ankara, Turkey; ^2^Department of Restorative Dentistry, Faculty of Dentistry, Kırıkkale University, Kırıkkale, Turkey

**Keywords:** Color, Coloring agent, Enamel microabrasion, Fluoride, Staining

## Abstract

**Background.** Enamel microabrasion can eliminate enamel irregularities and discoloration. This study evaluated the staining susceptibility of enamel after microabrasion using different clinical protocols.

**Methods.** A total of 210 extracted bovine incisors were used in this study. The teeth were randomly divided into five groups of 42 teeth each (n=42), including group 1: control (no treatment), group 2: microabrasion, group 3: microabrasion + fluoride polishing, group 4: macroabrasion (fine-grit diamond bur) + microabrasion, and group 5: macroabrasion (finegrit diamond bur) + microabrasion + fluoride polishing. The groups were then randomly divided into two subgroups for discoloration procedures: coffee and distilled water (n=21). A spectrophotometric evaluation was carried out at baseline and on the 1st, 7th, 14th, and 28th days of the staining period. Statistical analyses were performed using repeated-measures ANOVA and the post hoc Bonferroni test at a significance level of 0.05.

**Results.** The greatest color change was observed in group 2 specimens, which were immersed in coffee solutions. The color change values for groups 3, 4, and 5 specimens, which were immersed in distilled water, were higher than those in group 1 specimens (*P*<0.05). The fluoride polishing + enamel microabrasion treatment groups (groups 3 and 5) exhibited greater resistance to color changes than the matched non-fluoride-polished groups (groups 2 and 4) (*P*<0.05).

**Conclusions.** The teeth that underwent enamel microabrasion treatment and were polished with fluoride gel became more resistant to color changes. Our findings confirm that enamel microabrasion treatment is a conservative method for localized discoloration.

## Introduction

 Microabrasion is a treatment option for color differences and surface texture inconsistencies on teeth, especially in cases of superficial tooth stains, enamel decalcification, and minor defects.^1,2^ Microabrasion is also recognized as a safe, conservative, and non-restorative approach.^3^ In 1982, Pini et al prepared a mixture for microabrasion treatment by adding pumice to 36% hydrochloric acid.^4^ Croll et al^5^ proposed using the same mixture but with 18% hydrochloric acid because they were concerned about the acid content. According to Croll et al, an ideal microabrasion system has a low acid content and includes abrasive particles in a water-soluble mixture applied with a slowly rotating handpiece to minimize the spread of the compounds and ensure that the procedure is safe. Furthermore, to shorten the application time, Croll et al also recommended using an extra-fine diamond bur before applying microabrasion agents.^4,5^

 Contemporary microabrasion treatment is based on Croll’s concepts, a procedure involving the removal of a layer of enamel tissue. In previous studies, various microabrasion products have been used, and the depth abraded has been reported to vary from 20 to 200 µm, depending on the acid content and the application time.^3,6^ Acid erosion and the polishing impact of abrasive particles eliminate surface discoloration and produce a smoother surface by removing micropores, yielding a prism-free outer enamel surface following microabrasion treatment. This increases the esthetics of the enamel by brightening it, making it more reflective.^4,7^ Many studies have found enamel microabrasion to be an effective and conservative treatment.^6,8-10^ According to Sundfeld et al,^6^ 5‒10 applications of a microabrasion system can result in enamel erosion, which is tolerable under clinical conditions. According to a recent study,^11^ 120 seconds of microabrasion therapy reduces enamel thickness by approximately 10%, indicating that it is a safe and conservative approach. Depending on the severity of the enamel staining, the number of applications or techniques of application may vary. The degree of enamel staining also determines the quantity and technique of applying the microabrasion product. To minimize clinical hours, enamel macro-reduction can be performed by first using a tapered fine-diamond bur, followed by polishing the micro-abraded surface using felt disks and polishing paste or fluoride paste. Enamel microabrasion can also be used in combination with bleaching treatments.^4^

 Individuals with tooth enamel staining and discoloration typically visit dental clinics. The etiology of superficial enamel discoloration, which frequently results in esthetic concerns, tends to vary. It can be caused by stains from extrinsic sources, including coffee, tea, and cigarettes, or it may have an intrinsic etiology, e.g., amelogenesis imperfecta or tetracycline staining. Color abnormalities are frequently restored using direct or indirect restorative materials, which yield satisfactory esthetic outcomes. However, these restorations require long-term repair and recurring replacements, causing the tooth to enter the restorative cycle.^7^

 Previous research,^2,3,12^ including the studies mentioned earlier, focused on enamel loss and surface roughness attributed to the microabrasion technique. This technique increases the roughness of the enamel surface and is also related to reduced enamel microhardness. However, both effects can be reversed by the polishing procedure or saliva exposure.^4^Despite the benefits and resources available for this procedure, there is still a lack of knowledge about the outcomes of this strategy. Furthermore, there is little evidence that these treated surfaces will resist extrinsic discoloration if they are later exposed to extrinsic discoloring agents.

 Therefore, this in vitro study evaluated the staining susceptibility of enamel after different microabrasion application protocols after 28 days of immersion in distilled water and coffee. The null hypothesis tested was that microabrasion application protocols did not affect the staining susceptibility of enamel.

## Methods

###  Experimental design

 In this study, 210 sound bovine incisors without cracks or erosion were used as specimens that were cleaned and disinfected using 0.1% thymol. The required minimum sample size was calculated using G*Power 3.1 statistical software (Heinrich Heine, University of Dusseldorf, Dusseldorf, Germany). An alpha-type error of 0.05, an effect size of 0.25, and a beta power of 0.80 were specified, and the minimal estimated total sample size was calculated at n = 200. The roots of the incisors were removed, and the crowns were cut into 5-mm blocks with a diamond disk on a low-speed cutting machine (Isomet 1000, Buehler, Lake Bluff, IL, USA). With their labial surfaces exposed, the specimens were embedded in transparent autopolymerizing acrylic resin (Integra Self Cure Acrylic; Birlesik Grup Dental, Ankara, Turkey). Silicon carbide paper was not applied to the labial surfaces of the enamel. The specimens were randomly divided into five groups, and each group was randomly assigned to a microabrasion technique.

*Group 1:* Control: No treatment was applied to the enamel surface.

*Group 2:* Microabrasion: A water-soluble gel (Opalustre, Ultradent Products Inc., South Jordan, Utah, USA) containing mildly concentrated hydrochloric acid (6%) and a fine-grit silicon carbide abrasive paper was applied to the enamel surface using a rubber cup specially designed for a 500-rpm handpiece. The abrasive compound was applied in three applications^8,9^ of one minute each, with irrigation between each application.

*Group 3:* Microabrasion + fluoride polishing: The enamel microabrasion procedure was similar to group 2. After the abrasion compound treatment, a 2% neutral-pH sodium fluoride gel (Flugel, DFL Indústria e Comércio S.A. Rio de Janeiro, RJ, Brazil) was applied to the enamel surfaces for four minutes.

*Group 4:*Macroabrasion (fine-grit diamond bur) + microabrasion: Before administering the enamel microabrasion therapy, the superficial layer of the enamel surface was removed for 5–10 seconds with a high-speed fine-grit water-cooled tapered diamond bur (Hicare Co., Guangzhou, China). The enamel microabrasion procedure was similar to group 2.

*Group 5:* Macroabrasion (fine-grit diamond bur) + microabrasion + fluoride polishing: The enamel microabrasion procedure was similar to group 4. After the abrasion compound treatment, a 2% neutral-pH sodium fluoride gel (Flugel, DFL Indústria e Comércio S.A., Rio de Janeiro, Brazil) was applied to the enamel surfaces for four minutes.

 All the specimens were kept in distilled water for 24 hours. Following the application of the various enamel microabrasion procedures, the specimens in the five groups were randomly divided into two subgroups (n = 21) to undergo staining procedures.Coffee was used as the staining solution, and distilled water was used on the control subgroup. The coffee was made with two grams of Nescafe powder (Nestle, Vevey, Switzerland) and 200 mL of hot water. The solutions were refreshed every day.

###  Color measurements

 A spectrophotometer was used to determine the baseline color readings of all the specimens after the enamel microabrasion procedure (VITA Easyshade Compact, Vident, Germany). To remove background influences, each measurement was taken against a standard white background, and all the measurements were made at the same time of the day. While taking measurements, the instrument’s fiber-optic tip was set perpendicular to the specimens and parallel to the ground, and calibration was performed before each measurement. For each specimen, three measurements were taken. After determining the baseline color values, half of each experimental group’s specimens were immersed in distilled water and the other half in a coffee solution. Color measurements were taken on the 1st, 7th, 14th, and 28th days.^13^ The color change in the specimens was formulated with the ΔE parameter calculated using the values L, a, and b^14^:

 ΔE = [(ΔL0 − ΔL1)^2^ + (Δa0 − Δa1)^2^ + (Δb0 − Δb1)^2^]^1/2^

 All the restorative procedures and color measurements were performed by an operator on the research team. The acceptable limit of ΔE was established at 3.5 in this investigation.

###  Statistical analysis

 The data were analyzed using SPSS 22.0 (IBM, Chicago, IL). The mean and standard deviation were used in evaluating the color measurements using the descriptive statistical method. The data distribution was controlled with the Shapiro–Wilk test, and a normal distribution was observed. The repeated-measures analysis of variance (ANOVA) test was used to determine the differences between intragroup repeated measurements. Differences between more than two groups were analyzed using the post hoc Bonferroni test at a significance level of *P* < 0.05.

## Results

**Figure 1:**
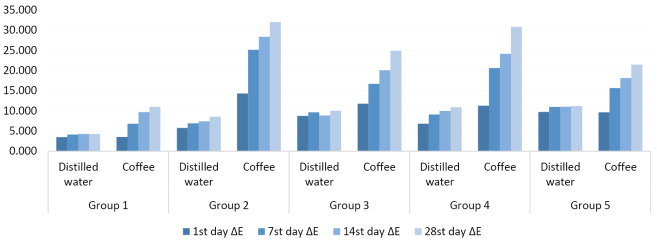
The color change values (ΔE) of the groups.

 The greatest color change was observed in group 2 specimens (i.e., microabrasion without fluoride polishing) (ΔE = 31.9 ± 0.8) immersed in a coffee solution. The group least affected by the discoloration treatment was group 1, i.e., the control group (ΔE = 3.4 ± 0.7), with specimens immersed in distilled water (Table 1 and Figure 1).

**Table 1:** Means (standard deviation) of changes in color parameters following the discoloration procedures after enamel microabrasion

**Color change values ( ΔE)**	**Group 1**		**Group 2**		**Group 3**		**Group 4**		**Group 5**	
	**Distilled water**	**Coffee**	**Distilled water**	**Coffee**	**Distilled water**	**Coffee**	**Distilled water**	**Coffee**	**Distilled water**	**Coffee**
1st day	3.4 ± 0.7	3.5 ± 0.7	5.7 ± 0.7	14.3 ± 0.6	8.7 ± 0.6	11.7 ± 0.6	6.8 ± 0.7	11.3 ± 0.7	9.7 ± 0.6	9.6 ± 0.6
7st day	4.1 ± 0.7	6.8 ± 0.8	6.9 ± 0.7	25.1 ± 0.7	9.6 ± 0.7	16.7 ± 0.7	9.1 ± 0.7	20.6 ± 0.8	11 ± 0.7	15.6 ± 0.7
14st day	4.2 ± 0.8	9.7 ± 0.9	7.4 ± 0.8	28.3 ± 0.8	8.8 ± 0.8	20.1 ± 0.8	9.9 ± 0.8	24.1 ± 0.8	11 ± 0.8	18.1 ± 0.8
28st day	4.3 ± 0.8	10.9 ± 0.9	8.5 ± 0.8	31.9 ± 0.8	10 ± 0.8	24.8 ± 0.8	10.9 ± 0.8	29.4 ± 0.8	11 ± 0.9	21.4 ± 0.8
*	-	1<7, 1<14, 1<28	1<28, 7<28	1<7, 1<14, 1<28 7<28, 14,<28	1<7, 7<28	1<7<14<28	1<7<14<28	1<7<14<28	1<28	1<7, 1<14, 1<28 7<28, 14,<28

* Statistically significant difference (*P* < 0.05)

 The color change values recorded for group 1 specimens (control), immersed in coffee, were higher than the values recorded in group 1 specimens (control), immersed in distilled water after 14 days and 28 days (*P* = 0.000; Table 2).

**Table 2:** Pairwise comparison of color change values

**Groups**	**Staining** **solutions**	** Color measurement ** ***P*** ** values**			
		**∆E _1**	**∆E _7**	**∆E _14**	**∆E _28**
Group 1: Control	Coffee	-	-	0.000	0.000
	Distilled water	-	-	-	-
Group 2: Microabrasion	Coffee	0.000	0.000	0.000	0.000
	Distilled water	-	-	-	-
Group 3: Microabrasion + fluoride polishing	Coffee	-	0.000	0.000	0.000
	Distilled water	-	-	-	-
Group 4: Macroabrasion (fine-grid-diamond bur) + microabrasion	Coffee	0.000	0.000	0.000	0.000
	Distilled water	-	-	-	-
Group 5: Macroabrasion (fine-grid-diamond bur) + microabrasion + fluoride polishing	Coffee	-	0.001	0.000	0.000
	Distilled water	-	-	-	-

(-) = no statistically significant difference (*P* > 0.05)

 The color change values recorded for the specimens of group 1 specimens, immersed in coffee, were lower than the values recorded for other coffee groups for all the immersion periods (*P* = 0.000; Table 1).

 The color change values recorded for group 2 specimens (microabrasion without fluoride polishing) and group 4 specimens (diamond bur + enamel microabrasion treatment without fluoride polishing), immersed in coffee, were higher than the values recorded for group 2 specimens (microabrasion without fluoride polishing) and group 4 specimens (diamond bur + enamel microabrasion treatment without fluoride polishing), which were immersed in distilled water for all periods (*P* = 0.000; Table 2).

 The color change values recorded for group 3 specimens (enamel microabrasion treatment with fluoride polishing) and group 5 specimens (diamond bur + enamel microabrasion treatment with fluoride polishing), immersed in coffee, were higher than the values recorded for group 3 specimens (enamel microabrasion treatment with fluoride polishing) and group 5 specimens (diamond bur + enamel microabrasion treatment with fluoride polishing), immersed in distilled water, after 7, 14, and 28 days (*P* = 0.000; Table 2).

 The color change values recorded for the specimens of groups 2 and 4 (microabrasion without fluoride polishing groups), immersed in coffee, were higher than the values recorded for groups 3 and 5 (microabrasion with fluoride polishing), immersed in coffee, after 7, 14, and 28 days (*P* < 0.05; Table 1).

**Figure 2:**
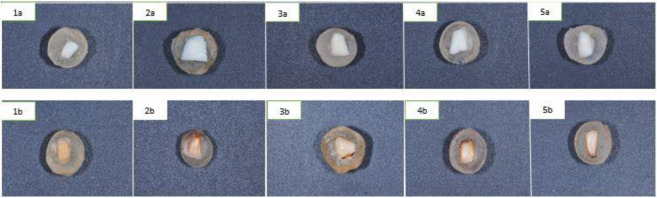
The color change images (ΔE) at the 28-day interval after immersion in coffee solution. 1 = control group, 2 = microabrasion, 3 = microabrasion + fluoride polishing, 4 = macroabrasion + microabrasion, 5 = macroabrasion + microabrasion + fluoride polishing, a = immersed in distilled water, b = immersed in coffee.

 The effects of staining progressively increased over time for all the groups except for group 1 specimens immersed in distilled water. Clinically unacceptable color differences were observed in all the groups except for group 1 specimens immersed in distilled water from one day of immersion in distilled water and coffee (ΔE ≥3.5) (Table 1). Figure 2 shows the representative specimen images of all the groups at baseline and after 28 days.

## Discussion

 The present study evaluated different clinical application protocols of microabrasion (enamel microabrasion/with or without fluoride polishing and macroabrasion + microabrasion/with or without fluoride polishing) and the subsequent staining susceptibility of the treated enamel in distilled water and coffee. Based on the results, microabrasion and macroabrasion + microabrasion with fluoride polishing resulted in better staining outcomes than the microabrasion treatment without fluoride polishing. Therefore, the null hypothesis was rejected.

 For homogeneous specimen allocation, bovine enamel was chosen as the test substrate in this study. Although using human enamel in dental material testing is preferable, it has been established that bovine enamel could be safely used as a substitute for human enamel, especially since a large crown size is required for preparing samples with similar crown sizes.^15,16^

 Microabrasion involves removing tooth surface discoloration by applying a material containing acid and abrasive particles to the enamel surface. This procedure causes minimal enamel loss and yields an enamel surface with significant homogeneity, smoothness, and gloss over time—clinically known as the *abrasion effect*.^8^ Loguercio et al^17^ examined two commercially available microabrasion treatments to eliminate fluorosis stains and reported that Opalustre was more effective than Prema. They attributed this finding to the potentially large size of the silica granules in Opalustre. Therefore, Opalustre was used in our study because it was the most readily available and most widely used microabrasion product. In addition, it contains less HCl and has larger silica granules than similar dental materials.

 Many factors affect the enamel surface after microabrasion treatment, including manual or mechanical processes used, the interval between applications, handpiece speed, the pressure applied, the type/concentration of acid, and abrasive particle type.^11^ Based on years of clinical use, in some severe cases, it has also been established that macroabrasion with a tapered, fine-diamond bur as the first step reduces the clinical hours required for the procedure.^8^ In this study, different clinical application protocols for enamel microabrasion treatment, including the macroabrasion step, were investigated, and the technical factors were standardized, including the number of applications, intervals, and pressure applied.

 Changes in the color components (L*, a*, and b*) were used to assess the resistance of the enamel to discoloration after undergoing microabrasion treatments.^16^ In this study, a spectrophotometer (VITA Easyshade) was used for standard color change quantification. In terms of sensitivity, reliability, reproducibility, and accuracy, the VITA Easyshade spectrophotometer has been validated in numerous studies.^18,19^ The CIEL*a*b* color system is used in the VITA Easyshade and is especially useful because it allows the user to specify how the color parameters change in the system.

 In this study, the specimens undergoing enamel microabrasion treatment without fluoride polishing (group 2) showed the most color changes by the 28th day after being immersed in coffee. Furthermore, specimens that underwent enamel microabrasion treatment without fluoride polishing, immersed in distilled water (group 2), exhibited greater color changes than the control group (group 1). This finding could be explained since the remaining enamel surface was slightly thinner and translucent, and the dentin seemed more evident following treatment on micro-abraded teeth; in addition, they might have a darker shade or yellowish coloration.^6,9^ Furthermore, in this study, different protocols of microabrasion followed by fluoride polishing (groups 3 and 5) exhibited greater resistance to staining than the unpolished groups (groups 2 and 4). After microabrasion treatment, polishing with fluoride reduced surface roughness and increased the resistance of the enamel surface to discolorations, affecting esthetic properties and clinical longevity. According to Fragoso et al,^20^ microabrasion followed by polishing with diamond paste or prophylactic fluoride paste increased hardness and enhanced surface smoothness of the enamel.

 A visible color change of 3.5 units is the standard in most clinical trials.^21^ The acceptable limit of ΔE was established at 3.5 in this investigation. Color change values (ΔE) > 3.5 units could be recognized with the naked eye in all stages of the assessment across all the experimental groups. In this study, the color change values (ΔE) in all the groups, including the control group, were above the acceptable threshold of 3.5. These findings can be explained because the color values of the teeth were high before the microabrasion treatment, and the teeth undergoing microabrasion exhibited reduced lightness.^9^

 In the dental literature, there was no study on color changes after enamel microabrasion treatment. Paic et al^3^ investigated the color changes of human enamel surfaces after using two microabrasion compounds (Prema and Opalustre) with different treatment periods of 10, 20, 30, and 40 seconds, respectively. According to Paic et al, there were no significant changes in the color measurements between the pastes during the treatment period. Jahanbin et al^22^ investigated the color change values of an artificially white spot lesion treated with two microabrasion techniques with five minutes of daily immersion in a tea-coffee solution for one week and found color change values (ΔE) above the acceptable threshold of 3.5 after enamel microabrasion treatment. Baĝlar et al^23^ evaluated the color change values for prototype microabrasion paste and Opalustre on fluorosis-stained enamel. After enamel microabrasion treatment, the color change values (ΔE) were higher than the acceptable threshold of 3.5. These findings are consistent with the results of the present study.

 As a staining solution, coffee was chosen for the present study due to its routine use in daily life and its potential to produce distinct color changes in esthetic restorative materials. Several studies have established that the average time spent drinking a cup of coffee is 15 minutes in a single day.^13^ For individuals who drink three glasses of coffee per day, on average, the 28-day holding duration used in this study is approximately equivalent to over two years of coffee consumption in real life.^13^

 One important limitation of the present study was that the specimens used were intact enamel surfaces without any superficial defect and discoloration (i.e., fluorosis and white spot lesions). However, it is difficult to determine whether the enamel removal via the microabrasion techniques performed would be sufficient to eliminate any enamel anomaly. Furthermore, the reduction in enamel thickness and surface roughness following the microabrasion procedures was not measured in this study. These parameters are useful for investigating the correlation with color changes. Another important limitation of the study was that the specimens were stored in distilled water instead of artificial saliva after the enamel microabrasion treatment. Enamel remineralization in artificial saliva would reduce any potential bias in the methodology. Another limitation of the study was that only two staining solutions were used, which is one of several factors that could influence the intensity of enamel staining. Finally, additional in vitro and in vivo research to address these shortcomings may be needed to corroborate the conclusions of this study.

## Conclusion

 Within the limitations of this study, the following conclusions can be drawn:

- The greatest color change was observed in specimens that underwent enamel microabrasion treatment without fluoride polishing and were then immersed in a coffee solution.
- Specimens that underwent enamel microabrasion treatment in combination with polishing with fluoride became more resistant to color changes.
- In comparison to direct and indirect restorations, enamel microabrasion therapy appears to be a conservative approach, despite the limitations of this in vitro study.

## Authors’ Contribution

 BHS, HM, and ÇC contributed to the study concept and design. BHS, HM, and ÇC contributed to material preparation and data collection and analysis. All the authors commented on previous versions of the manuscript. All the authors read and approved the final manuscript.

## Funding

 None.

## Ethics Approval

 Based on Kirikkale university animal experiments local ethics committee directive, we did not treat or extract teeth on any animals for this study. We only received samples to be thrown away at the slaughterhouse.

## Competing Interests

 The authors do not have any financial interest in the companies whose materials are included in this article.
